# The Ubp3/Bre5 deubiquitylation complex modulates COPII vesicle formation

**DOI:** 10.1111/tra.12766

**Published:** 2020-10-20

**Authors:** Natalia Gomez‐Navarro, Jérôme Boulanger, Elizabeth A. Miller

**Affiliations:** ^1^ Cell Biology Division MRC Laboratory of Molecular Biology Cambridge UK

**Keywords:** Bre5, COPII, ER export quality control, Kar2, Ubp3

## Abstract

The appropriate delivery of secretory proteins to the correct subcellular destination is an essential cellular process. In the endoplasmic reticulum (ER), secretory proteins are captured into COPII vesicles that generally exclude ER resident proteins and misfolded proteins. We previously characterized a collection of yeast mutants that fail to enforce this sorting stringency and improperly secrete the ER chaperone, Kar2 (Copic et al., *Genetics* 2009). Here, we used the *emp24Δ* mutant strain that secretes Kar2 to identify candidate proteins that might regulate ER export, reasoning that loss of regulatory proteins would restore sorting stringency. We find that loss of the deubiquitylation complex Ubp3/Bre5 reverses all of the known phenotypes of the *emp24Δ* mutant, and similarly reverses Kar2 secretion of many other ER retention mutants. Based on a combination of genetic interactions and live cell imaging, we conclude that Ubp3 and Bre5 modulate COPII coat assembly at ER exit sites. Therefore, we propose that Ubp3/Bre5 influences the rate of vesicle formation from the ER that in turn can impact ER quality control events.

## Graphical abstract

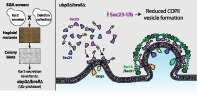

The ubiquitin protease complex Ubp3/Bre5 stabilizes the COPII subunit Sec23 through direct deubiquitylation. Using biochemistry, genetics, and live‐cell imaging, we observe that Ubp3/Bre5 modulates COPII coat assembly at ER exit sites. We propose that Ubp3/Bre5 influences the rate of vesicle formation from the ER that in turn impacts ER quality control. This work highlights how post‐translational modifications impact COPII coat function and regulate the secretory pathway.

## 1 INTRODUCTION

Vesicle formation from the endoplasmic reticulum (ER) represents a central cellular quality control checkpoint. As COPII vesicles form at the ER membrane, they select cargo for enrichment into the emerging bud. For the most part, these cargo proteins are mature, folded proteins that have been released from the abundant ER chaperones that promote their folding and assembly. Such selection for folded cargo ensures that aberrant and misfolded proteins are not released to downstream compartments, where they may trigger aggregation and interrupt cell function. However, non‐selective capture of nascent proteins can also occur, in a process known as bulk flow.^1^, ^2^ Indeed, non‐specific leakage of ER resident proteins necessitates an ER retrieval pathway that recognizes escaped ER residents and returns them to the ER via COPI‐coated vesicles.

We previously investigated the mechanisms that contribute to ER retention using a genetic screen that identified yeast mutants that have lax ER retention.^3^ The basis for the screen was detection of the abundant ER lumenal chaperone, Kar2, at the cell surface. Kar2 is normally efficiently retained within the cell by the combination of ER sorting stringency preventing capture into COPII vesicles and efficient ER retrieval via the KDEL‐receptor, Erd2, to collect any escaped protein. We reasoned that if ER leakage increases, then the retrieval pathway may become overwhelmed, resulting in release of Kar2 to the cell surface, which we detected using colony immunoblotting methods. This screen resulted in the identification of 87 mutants that impact various secretory pathway functions to yield an increase in Kar2 secretion.^3^

One of the top hits in our screen was the p24 family of proteins, which function as export receptors for GPI‐anchored proteins (GPI‐APs). Absence of p24 proteins and the resulting reduction in GPI‐AP packaging into COPII vesicles has multiple effects.^4^, ^5^ First, the local membrane bending energy of the ER seems to be reduced such that the bilayer can be deformed without structural rigidity conferred by the outer COPII coat scaffolding protein, Sec13.^6^ Second, the space created in the vesicle by the lack of GPI‐AP capture causes an increase in the non‐specific bulk flow leakage of secretory proteins, misfolded proteins, and ER residents.^7^ Increased bulk flow is the likely cause of Kar2 secretion; the retrieval pathway is still intact in p24 mutants, but retrieval capacity is outweighed by ER leakage. Adding a second mutation that decreases the size of COPII vesicles to the p24 mutants reversed both Kar2 secretion and increased bulk flow phenotypes, confirming the model that steric crowding plays a central role in ER sorting stringency.^7^ Finally, cells lacking p24 proteins show constitutive activation of the unfolded protein response (UPR), likely as a result of chaperone depletion.^5^

Here, we sought to use the Kar2 secretion phenotype as a tool to identify novel factors that impact COPII vesicle formation and/or architecture. We performed a genome‐wide screen for mutants that reverse the Kar2 secretion associated with loss of the p24 protein, Emp24. We identify the ubiquitin protease complex, Ubp3/Bre5, as factors that impact vesicle formation from the ER. Loss of either Ubp3 or Bre5 reverses Kar2 secretion in many different mutant backgrounds, and also alleviates other phenotypes associated with loss of the p24 proteins. We propose that by modulating the ubiquitination state of the COPII subunit, Sec23, Ubp3, and Bre5 influence the rate of vesicle formation at the ER, and thereby contribute to sorting stringency.

## 2 RESULTS

### 2.1 A high‐throughput screen shows multiple pathways that reverse aberrant Kar2 secretion

**FIGURE 1:**
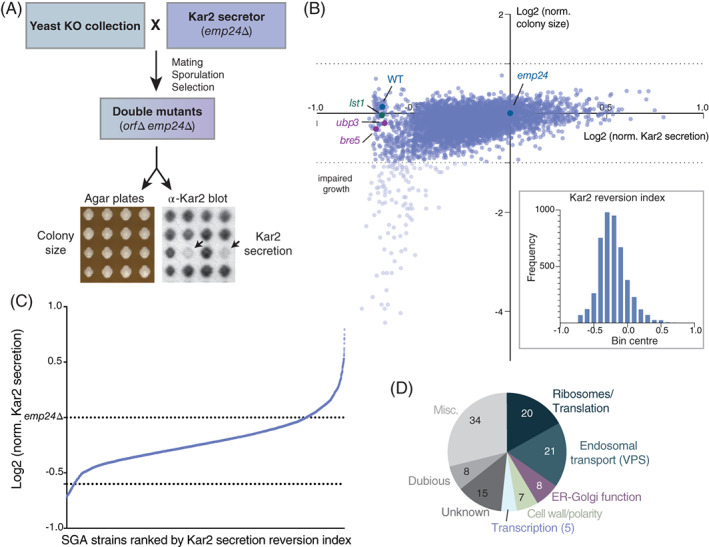
Genome‐wide analysis shows multiple pathways that reverse Kar2 secretion in an *emp24Δ* strain. A, Outline of the SGA procedure for generating a yeast double mutant library and subsequent analysis of Kar2 secretion. The arrows show examples of haploid double mutants that revert the Kar2 secretion phenotype. B, Each haploid double mutant strain was measured and normalized for colony size (y‐axis) and Kar2 secretion (x‐axis); values are plotted on a log2 scale. The wild‐type strain (dark blue) and the double mutant strains: *emp24Δ trp1Δ* (dark blue), *emp24Δ lst1Δ* (green), *emp24Δ ubp3Δ* and *emp24Δ bre5Δ* (purple) are shown in the scatter plot. The histogram in the inset represents the distribution of the Kar2 reversion index in the double mutant library. C, Strains were ranked according to Kar2 secretion reversion index. A threshold to define double mutants that revert Kar2 secretion was set to −0.6. D, Functional classification of the Kar2 secretion revertants identified in the screen. The number of genes identified in each category is shown

In order to identify factors that influence ER sorting, we used synthetic genetic array (SGA) methodology to survey the yeast genome for mutants that reverse the Kar2 secretion phenotype of a p24 mutant. An *emp24Δ* query strain was crossed to the yeast deletion collection, and haploid double mutants containing the *emp24Δ* deletion plus deletion of a second gene were recovered (Figure 1A). Among the 4854 deletion strains in the collection, 72 strains did not yield haploid double mutants after the SGA procedure, likely because of synthetic sick/lethal interactions. Colony size and Kar2 secretion were measured in the resulting library of 4782 haploid double mutant strains, and normalized to that of an *emp24Δ trp1Δ* double mutant. This control strain had undergone the SGA procedure, so had the same resultant genetic background as the library, and showed Kar2 secretion levels similar to the *emp24Δ* parental strain (Table S1). Deletion of 110 genes resulted in impaired growth of cells in the *emp24Δ* background (Figure 1B). These double mutants were excluded from the Kar2 secretion analysis because growth defects confound analysis of Kar2 levels from colony overlay immunoblots. We calculated a Kar2 reversion index, which is the log2 ratio of the Kar2 secretion signal in a haploid double mutant relative to the *emp24Δ trp1Δ* mutant (Figure 1B). Considering only double mutants with normal growth, the cohort of mutants with reduced Kar2 secretion included the *emp24Δ lst1Δ* mutant that we previously demonstrated reversed Kar2 secretion to wild‐type levels (Figure 1B).

Analysis of the Kar2 reversion indices across the double mutant library showed a mean reversion of −0.2 (Figure 1B, inset) with a SD of 0.2. Using 2 standard deviations (ie, ‐0.6) as a cutoff threshold we identified 118 mutants with Kar2 secretion levels reduced relative to that of *emp24*Δ cells (Figure 1C, Table S1). In order to classify the Kar2 secretion revertants we analyzed the biological processes in which they are involved. We observed GO‐term enrichment for proteins involved in ribosome function/biogenesis and translation, and in endosomal trafficking. Additional categories that were not necessarily GO‐enriched included proteins involved in ER‐Golgi function, cell wall function and cell polarity, and transcription (Figure 1D, Table S1). Because of our interest in ER quality control, we focused on the ER‐Golgi function hits, and specifically on two members of a known complex, Ubp3 and Bre5.

### 2.2 The absence of the Ubp3/Bre5 complex reverts Kar2 secretion as a result of a reduction in ER‐to‐Golgi transport

**FIGURE 2:**
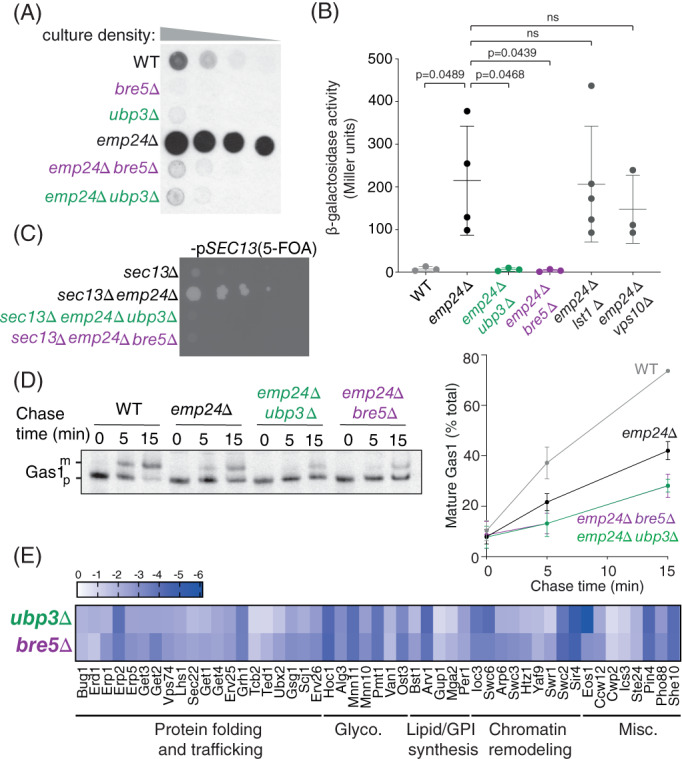
The absence of the Ubp3/Bre5 complex reverts Kar2 secretion as a result of a reduction in ER‐to‐Golgi transport. A, Extracellular Kar2 was detected by colony overlay immunoblot in the indicated yeast strains. Serial dilutions of cells at stationary phase were spotted onto YPD plates. After ~5 hours growth, colonies were overlaid with nitrocellulose and secreted Kar2 detected with Kar2‐specific antibodies. B, Exponentially growing wild‐type and mutant cells carrying a *UPRE*‐*LacZ* reporter gene were lysed, and ß‐galactosidase activity measured using ONPG as a substrate. Error bars depict mean and SD of at least three independent experiments. Statistical test was a one‐way ANOVA with Dunnett's multiple comparisons test. C, The indicated strains were grown to exponential phase, serially diluted and spotted on media containing 5‐fluoroorotic acid (5‐FOA) to counter‐select for the wild‐type p*SEC13*::*URA3* plasmid. D, Wild type, *emp24Δ, emp24Δ ubp3Δ* and *emp24Δ bre5Δ* cells were subjected to pulse‐chase analysis with [^35^S] methionine. Gas1 was immunoprecipitated from cell lysates at the indicated times. Immunoprecipitated proteins were detected by SDS‐PAGE/autoradiography (left panel). Maturation of Gas1 was quantified by phosphorimage analysis (right panel) (E) Heat‐map showing the Kar2 reversion index of *ubp3Δ* and *bre5Δ* in different Kar2 secretor strains involved in a wide variety of biological processes

Ubp3 and Bre5 form a deubiquitylation complex that acts directly on Sec23 to regulate its turnover.^8^, ^9^ To confirm the results from our SGA screening, *ubp3Δ* and *bre5Δ* deletions were introduced by PCR‐mediated integration into an *emp24Δ* strain, and Kar2 secretion measured by colony immunoblot. Indeed, Kar2 secretion was reversed by both the *UBP3* and *BRE5* deletions (Figure 2A). We next asked if the absence of the Ubp3/Bre5 complex could also rescue other phenotypes associated with Emp24 loss, including induction of the unfolded protein response (UPR), and bypass‐of‐Sec13.^4^, ^5^, ^10^ To measure UPR activation, we introduced a UPRE‐LacZ reporter plasmid^11^ into wild‐type and mutant strains, and quantified β‐galactosidase activity. As previously reported, the *emp24Δ* mutant had high levels of constitutive UPR, which was reversed by additional deletion of either *UBP3* or *BRE5* (Figure 2B). This reversal suggests that UPR activation in *emp24Δ* cells is primarily caused by increased export of ER chaperones, such that reversal of leakage conferred by loss of Ubp3/Bre5 alleviates ER stress, resulting in reduced activation of UPR. In contrast, UPR activation in the *emp24Δ* background was unaffected by deletion of two other reversion mutants, *vps10Δ*, and *lst1Δ* (Figure 2B), although both the *vps10Δ* and *lst1Δ* single mutants also have constitutive UPR (Supplemental Figure 1).

Emp24 loss creates permissive conditions that result in a bypass‐of‐sec‐thirteen (*bst)* phenotype, in which the normally essential COPII subunit Sec13 can be deleted.^4^, ^6^, ^10^ We therefore tested whether Sec13 bypass was reversed by the absence of the Ubp3‐Bre5 complex. We combined a *sec13Δ emp24Δ* strain with deletions in *UBP3* or *BRE5* and tested for viability on media containing 5‐FOA, which counter‐selects for *SEC13* on a *URA3*‐marked plasmid. The *bst* phenotype of the *emp24Δ* strain was indeed reversed by *UBP3* and *BRE5* deletions (Figure 2C). This observation supports the idea that the Ubp3/Bre5 complex promotes COPII vesicle formation, such that the absence of cargo during vesicle formation is no longer sufficient to provide viability when Ubp3/Bre5 function is lacking.

One trivial explanation for the multiple phenotypic reversions we observed upon loss of Ubp3/Bre5 is that defective ER export of GPI‐APs in the *emp24Δ* background is restored. However, when we examined the maturation of the GPI‐AP, Gas1, in *emp24Δ* and double mutant strains, we observed that GPI‐AP transport is not rescued (Figure 2D). Finally, we asked whether the reversion effects of Ubp3/Bre5 loss were unique to the *emp24Δ* condition or whether their deletion would similarly revert other Kar2 secretion mutants. We used the SGA method to introduce *ubp3Δ* and *bre5Δ* deletions into a set of 49 Kar2 secretion mutants that we previously identified,^3^ and measured Kar2 secretion by colony immunoblot. In each case, loss of Ubp3/Bre5 reduced Kar2 secretion observed in the single mutants (Figure 2E), suggesting a broad cellular effect rather than a function specific to the p24 pathway. Taken together, our analysis of the reversion of multiple *emp24Δ* phenotypes and the broad effect of Ubp3/Bre5 loss on other mutants supports the notion that the absence of Ubp3‐Bre5 complex causes a general reduction in ER‐to‐Golgi transport that in turn reduces improper leakage of ER residents.

### 2.3 UBP3 and BRE5 interact genetically with COPII coat subunits

**FIGURE 3:**
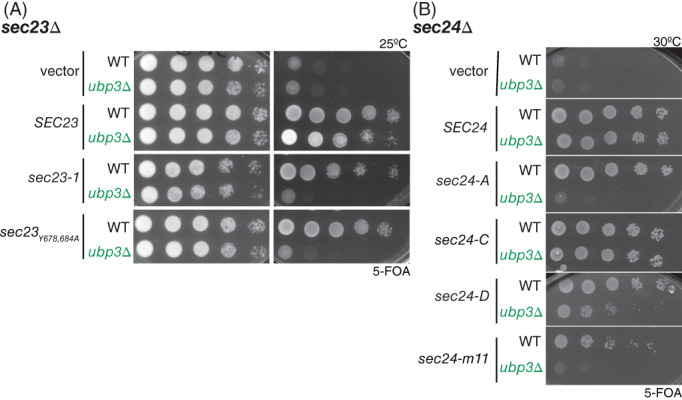
Genetic interactions between COPII proteins and Ubp3/Bre5. A, Plasmids encoding *SEC23* mutants ‐ *sec23‐1* and *sec23‐Y678,684A* ‐ were introduced into *sec23Δ* and *sec23Δ ubp3Δ* as indicated and grown on 5‐FOA at 25°C. B, Plasmids encoding *SEC24* mutants ‐ *sec24‐A, sec24‐C, sec24‐D* and *sec24‐m11* ‐ were introduced into *sec24Δ* and *sec24Δ ubp3Δ* as indicated and grown on 5‐FOA at 30°C

The Ubp3/Bre5 complex stabilizes the COPII subunit Sec23 through direct deubiquitylation, which prevents its proteasomal degradation.^8^, ^9^ Given the function of Sec23 in vesicle formation and its stabilization by Ubp3/Bre5, we tested whether deleting *UBP3* in strains expressing mutant versions of Sec23 impacts viability. To examine the genetic interactions, a chromosomal deletion of *UBP3* was introduced into a *sec23Δ* null strain carrying a p*SEC23‐URA3* plasmid. The resultant *sec23Δ ubp3Δ* strain was transformed with plasmids carrying WT or mutant forms of *SEC23*. The resulting transformants were tested for viability on media containing 5‐FOA, which counter‐selects for the *SEC23*::*URA3* plasmid. Both the temperature‐sensitive *sec23‐1* allele (S383L^12^) and mutations in the Sec31‐binding gelsolin domain (Y678,684A^13^) are permissive for growth as the sole copy of Sec23, but when Ubp3 is absent, cells carrying these mutations are inviable (Figure 3A). We also tested whether deletion of *UBP3* impacts the growth phenotypes of the Sec23 partner, Sec24, which is the cargo adaptor protein of the COPII coat. Deletion of *UBP3* in the context of various *SEC24* alleles showed distinct effects. Cargo binding mutants had variable phenotypes in the absence of Ubp3, with the A‐ and D‐site mutants reduced in viability but the C‐site mutant unaffected (Figure 3B). The *sec24‐m11* mutant, which impacts coat assembly and turnover^14^ was also inviable when *UBP3* was deleted (Figure 3B). Our interpretation of these genetic interactions is that Ubp3/Bre5 are required to maintain vesicle formation in the context of partially dysfunctional COPII coat proteins.

### 2.4 Loss of Ubp3/Bre5 affects COPII coat dynamics

**FIGURE 4:**
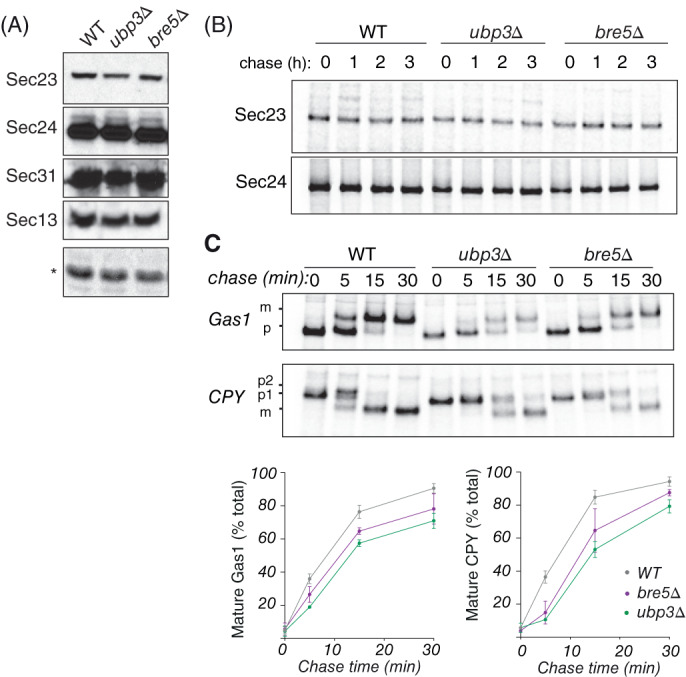
Ubp3/Bre5 mutants have secretory defects but normal COPII levels. A, COPII protein levels were analyzed in cell lysates prepared from equal numbers of exponentially growing cells of the indicated strains using immunoblot. Asterisk indicates unspecific band. B, Pulse‐chase analysis was used to determine the half‐lives of Sec23 and Sec24 in the indicated strains. After starvation cells were labeled with [^35^S] methionine and chased for the indicated times. Sec23 and Sec24 were precipitated from cell lysates using specific antibodies. Immunoprecipitated proteins were detected by SDS‐PAGE and autoradiography. C, Gas1 and CPY maturation were examined in wild‐type, *ubp3Δ* and *bre5Δ* strains by pulse‐chase with [^35^S] methionine. Gas1 and CPY were immunoprecipitated from lysates at the indicated times and were detected by SDS‐PAGE and autoradiography. The percent maturation from precursor (p) to mature (m) forms was quantified and plotted using Prism software. Error bars represent SD; n = 3

Because the Ubp3/Bre5 complex has previously been defined as a modulator of Sec23 stability, we measured Sec23 steady state levels (Figure 4A), and Sec23 turnover (Figure 4B) in the presence and absence of Ubp3 and Bre5. In our hands, loss of Ubp3/Bre5 did not affect the stability of Sec23 or other COPII proteins. We also did not detect the ubiquitinated form of Sec23, which was previously observed.^9^ These differences may reflect the different antibodies used in the different studies. In pulse‐chase experiments to measure secretion rates for two proteins, Gas1 and CPY, we recapitulated previous observations that secretion was modestly impaired in both the *ubp3Δ* and *bre5Δ* mutants, consistent with reduced COPII abundance (Figure 4B).

**FIGURE 5:**
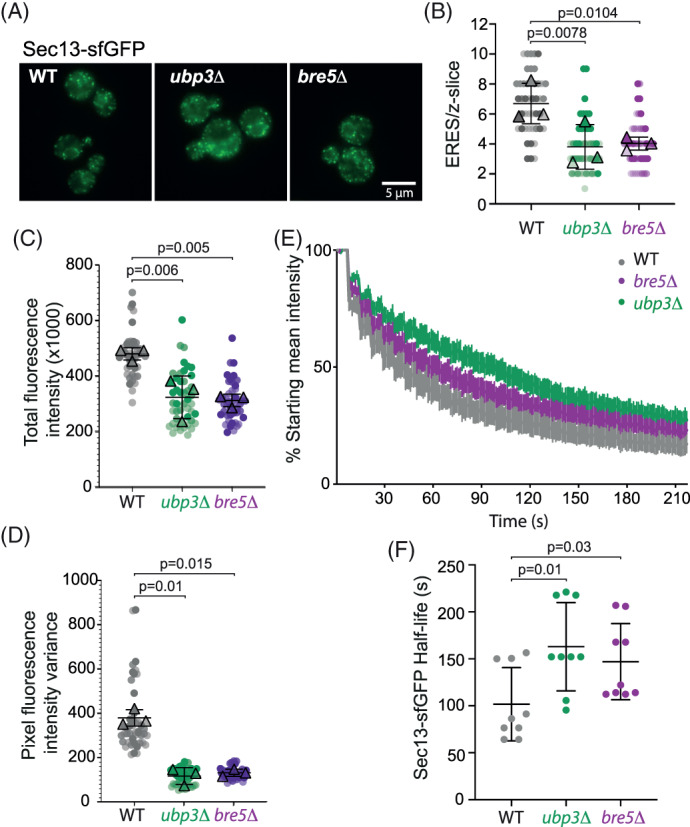
Loss of Ubp3/Bre5 reduces ERES number and COPII turnover. A, Sec13‐sfGFP shows broadly normal ER exit sites in wild‐type, *ubp3Δ* and *bre5Δ* cells. Scale bar 5 μm. B, Quantification of the number of ERES in the indicated strains expressing Sec13‐sfGFP. Values are the number of exit sites identified in a single confocal image plane in the center of a yeast cell. A total of 45 cells were measured in 3 independent experiments. Error bars represent SD; statistical test was a repeated measures one‐way ANOVA with Dunnett's multiple comparison testing. C, Total Sec13‐sfGFP fluorescence was measured in the same confocal image plane of the cell. A total of 45 cells were measured in three independent experiments. Error bars represent SD; statistical test was a repeated measures one‐way ANOVA with Dunnett's multiple comparison testing. D, Pixel fluorescence intensity variance, a measurement of the difference between the brightest foci and the dimmest background signal, was obtained for the indicated strains. A total of 45 cells were measured in three independent experiments. Error bars represent SD; statistical test was a repeated measures one‐way ANOVA with Dunnett's multiple comparison testing. E, Sec13‐sfGFP turnover at ER exit sites was quantified using fluorescence loss in photobleaching (FLIP). A small region of interest was repeatedly photobleached whilst total cellular fluorescence was measured over time. Error bars represent SEM; n = 9. (F) Sec13‐sfGFP half‐life in individual cells was measured in the indicated strains. Error bars represent SD; n = 9. Statistical test was an unpaired one‐way ANOVA

Previous morphological analysis of *ubp3Δ* and *bre5Δ* mutant strains showed accumulation of ER membranes consistent with reduced COPII vesicle formation.^9^ We sought to more directly interrogate COPII function by visualizing COPII marked ER exit sites. Deletion of *UBP3* or *BRE5* had no obvious effect on the localization of Sec13‐sfGFP at ER exit sites (Figure 5A), although the number of ERES was reduced in both the *ubp3Δ* and *bre5Δ* strains (Figure 5B). Moreover, the total fluorescent signal for Sec13‐sfGFP was reduced in the *ubp3Δ* and *bre5Δ* strains relative to wild‐type (Figure 5C), suggesting that coat stability is reduced in these cells. Similarly, Sec13‐sfGFP pixel fluorescence intensity variance was reduced in the *ubp3Δ* and *bre5Δ* mutants (Figure 5D), which indicates a slighly more homogeneous distribution between ERES and a cytoplasmic pool.^14^, ^15^ Together, these fluorescence measurements suggest that abundance and intracellular distribution of the COPII coat is subtly altered in the absence of Ubp3/Bre5.

To further define the effect of the absence of the Ubp3/Bre5 on COPII coat dynamics we performed fluorescence loss in photobleaching (FLIP) measurements of Sec31‐sfGFP at individual ERES (Figure 5E). We continuously bleached an area of the cell and measured the rate at which cellular fluorescence in an area outside the bleached region decayed. This is an indirect measure of the dynamics of the COPII coat cycling off the ER membrane and thereby passing through the bleached region of the cell. Fluorescence loss measurements showed reduced turnover of Sec13‐sfGFP in *ubp3Δ* and *bre5Δ* cells (Figure 5E,F). Increased lifetime of COPII proteins at ER exit sites is consistent with a reduction in the number of vesicles released from the ER.^14^

## 3 DISCUSSION

By capitalizing on the phenotype of improper Kar2 secretion, we sought to identify machinery that regulates formation of COPII vesicles. A genome‐wide screen for mutants that reverse Kar2 secretion in a p24 mutant background revealed a number of candidate regulators of membrane traffic. We recovered *LST1/SFB3* as a reversion mutant, validating our approach and confirming our previous findings that reduction of vesicle size reverses non‐specific leakage of Kar2 from the ER. Among the hits, one of the largest cohorts were proteins involved in vacuolar protein sorting. How traffic between the Golgi and vacuole impacts Kar2 leakage from the ER is unclear. One speculative model is that when vacuolar proteins are improperly secreted, Kar2 that has leaked from the ER can instead access vacuole‐bound vesicles and its release to the cell surface is thereby diminished. Alternatively, impairing vacuolar protein sorting may indirectly impact ER export by altering the flux of membrane and protein within the secretory pathway. Numerous *vps* mutants have constitutive UPR,^16^ which might link ER homeostasis to the reversal effects that we observe.

In focusing on likely regulators of COPII function, we further characterized the Ubp3/Bre5 deubiquitination complex, which acts directly on Sec23 to manage its turnover.^9^ In the context of lax protein quality control driven by loss of p24 proteins, additional loss of Ubp3/Bre5 most likely reduces Kar2 secretion simply by reducing the frequency of vesicle formation. The slow‐down in Gas1 and CPY maturation we observed in the *ubp3Δ* and *bre5Δ* strains is suggestive of a general reduction in ER export efficiency. If fewer vesicles are made, COPII turnover at ER exit sites will be reduced, again consistent with the phenotype we observed in the absence of Ubp3/Bre5. Presumably the rate of vesicle formation in the *ubp3Δ/bre5Δ* mutants is not reduced to such an extent that sufficient cargo accumulates to trigger the UPR, which is not constitutively active in these strains. We propose that a reduction in vesicle formation associated with loss of Ubp3/Bre5 can explain the reversal of all phenotypes associated with p24 loss. With the number of vesicles reduced, cargo will out‐compete ER residents for access to those vesicles, thereby reducing bulk flow and restoring ER sorting stringency. With vesicles again full of cargo proteins, the membrane bending energy at ER exit sites will again be high, requiring rigidity conferred by Sec13 and thus reversing the *bst* phenotype. Finally, if Kar2 leakage is prevented, then the likelihood of a constitutive ER stress condition is reduced, again consistent with the observed reduction in UPR. Therefore, we propose that Ubp3/Bre5 play an important role in modulating the rate of vesicle formation from the ER that in turn can impact ER quality control events.

Reduction in vesicle formation would explain the genetic interactions we and others have observed for the *ubp3Δ* and *bre5Δ* strains. Bre5 was first linked to ER export via a genetic interaction with the COPII subunit Lst1/Sfb3.^9^ We report multiple synthetic interactions with different alleles of Sec23 and Sec24 that impact distinct functions within the COPII coat. Moreover, high throughput analyses have showed numerous negative genetic interactions between *ubp3Δ* and *bre5Δ* and proteins that function in a variety of secretory events (thebiogrid.org^17^, ^18^). These broad genetic interactions are again consistent with reduced traffic from the ER: a deficit in COPII function or in secretory pathway function can be tolerated unless the absence of Ubp3/Bre5 further reduces flow of membrane and protein. However, because Ubp3/Bre5 also acts on COPI coat subunits,^19^ the pleiomorphic effects of loss of this deubiquitylating complex with regard to the secretory pathway are difficult to parse genetically. Biochemical analysis of COPII coat proteins and their function when deubiquitylation is abrogated will be required to fully understand the mechanism of reduced ER export.

Surprisingly, in our hands, Sec23 was not reduced in abundance or stability in the *ubp3Δ* or *bre5Δ* backgrounds as previously reported.^9^ However, ER exit site number, and COPII coat distribution and dynamics were clearly reduced. This leads us to suggest that ubiquitination of Sec23 modulates its functionality within the COPII coat in addition to impacting coat turnover. The ubiquitinated form of Sec23 does not co‐precipitate with Sec24,^9^ raising the possibility that deletion of Ubp3/Bre5 simply reduces the functionally available pool of Sec23. However, free Sec23 can still bind to various coat proteins, and can stimulate GTPase activity without the need for Sec24.^20^ Moreover, excess Sec23 is toxic to cells, presumably because it can trigger GTP hydrolysis on Sar1 without coupling to cargo selection. Thus, if free Sec23 accumulates in the *ubp3Δ/bre5Δ* condition, one would expect increased coat turnover rather than a more stable coat. Instead, ubiquitination of Sec23 may have more direct effect on how the coat subunits interact with each other. Ubiquitination of human Sec31 influences coat function via unknown mechanisms.^21^ Sar1 is also ubiquitinated (ptmfunc.com), although the functional relevance of this modification remains untested. Clearly, COPII coat ubiquitination, among other post‐translational modifications, warrants further investigation with regard to coat assembly and dynamics.

## 4 MATERIALS AND METHODS

### 4.1 Strains and plasmids

Yeast cultures were grown at 30°C in standard rich medium (YPD: 1% yeast extract, 2% peptone, and 2% glucose) or synthetic complete medium (SC: 0.67% yeast nitrogen base and 2% glucose supplemented with amino acids as needed). Strains (Table S2) were made by PCR‐based integration of auxotrophic or drug‐resistance markers. The plasmids (Table S3) were generated using standard cloning techniques.

### 4.2 Synthetic genetic array

SGA was performed as previously described^22^. Briefly, using a RoToR bench‐top colony arrayer (*Singer Instruments*) to manipulate libraries, the Mat‐a deletion collection was inoculated from glycerol stocks in a 384 well format and grown for 36 to 48 hours in YPD. The deletion collection was then crossed with the Mat‐alpha *emp24*::*NatMX* query strain. Mating was carried out on YPD plates followed by diploid selection in YPD containing G418 (200 ug/mL) and ClonNat (100 ug/mL). Cells were then sporulated at 25°C for 1 week on nitrogen starvation plates, before two rounds of transfer to haploid double mutant selection media containing all selection markers alongside the toxic amino acid derivatives canavanine and thialysine (*Sigma‐Aldrich*) to select against remaining diploids. SGA of mini‐libraries of Kar2 secretors were performed as described above crossing the Mat‐a Kar2 secretor mini‐library with the correspondent Mat‐alpha query strain (*ubp3*::*NatMX* or *bre5::NatMX)*.

### 4.3 Genome‐wide colony size measurements and quantification of Kar2 secretion

Haploid double mutant cell arrays were spotted to YPD plates using a 384‐pin tool and grown overnight. Colony size was measured using the PhenoBooth Colony Counter (*Singer Instruments*). Cells were then overlaid with nitrocellulose filters and incubated for a further 1 hour. Nitrocellulose filters were washed, blocked and incubated with α‐Kar2 polyclonal sera (provided by Randy Schekman, UC Berkeley). Secreted Kar2 was detected with HRP‐conjugated goat‐anti‐rabbit antibodies followed by ECL detection. Mean Kar2 secretion intensity was determined for each haploid double mutant using a bespoke Fiji script. During script execution, the user was first asked to draw a parallelepiped whose vertices pass through the center of the four corner spots, and then to define a circle of the size of one spot. The mean intensity within spots regularly spaced on a grid defined by the edges of the parallelepiped was then automatically measured and reported in a table.

### 4.4 Kar2 secretion assays

Serial dilutions of logarithmic phase cultures were spotted onto YPD plates and incubated at 30°C for 5 hours, at which point colonies were overlaid with nitrocellulose filters and incubated for a further 1 hour. Nitrocellulose filters were washed, blocked and incubated with α‐Kar2 polyclonal sera (provided by Randy Schekman, UC Berkeley). Secreted Kar2 was detected with HRP‐conjugated goat‐anti‐rabbit antibodies followed by ECL detection (Pierce, Rockford, Illinois).

### 4.5 β‐galactosidase assays

To determine β‐galactosidase activity of yeast cells carrying the UPRE‐LacZ reporter, exponentially growing cells were lysed in Buffer Z (60 mM Na_2_HPO_4_, 40 mM NaH_2_PO_4_, 10 mM KCl, 1 mM MgSO_4_, 50 mM 2‐mercaptoethanol) supplemented with 0.001% SDS and 2.5% chloroform. Lysates were incubated at 30°C for 15 minutes and the reaction was initiated with the addition of the substrate 2‐Nitrophenyl β‐D‐galactopyranoside (Thermo Scientific). When the mixture acquired a pale yellow colour the reaction was terminated by the addition of Na_2_CO_3_ and the absorbance at 420 nm and 550 nm was measured. Miller units were calculated according to the following formula: Miller Units = 1000 x (A420 ‐ (1.75 x A550)]/[Reaction time x Volume x OD600).

### 4.6 Serial dilutions

To observe growth in the absence of Sec13 and growth of strains carrying Sec23 or Sec24 mutations, cells were grown to saturation at 30°C in media lacking uracil. Serial dilutions (1:10) were made from the saturated cultures and spotted onto solid media without (control) or with 0.1% 5‐Fluoroorotic acid (5‐FOA, BioVision) to counter‐select for the corresponding URA3 plasmid (p*SEC13‐URA3*, p*SEC23‐URA3* or p*SEC24‐URA3*). Plates were scanned at day 2 or day 3 after spotting and growth at 30°C.

### 4.7 Western blot analysis

Protein extracts prepared by alkaline lysis of exponentially growing yeast were separated by SDS‐PAGE. Sec23, Sec24, Sec13, and Sec31 proteins were detected with the corresponding specific antibodies (provided by Randy Schekman, UC Berkeley). Steady state levels of proteins were then detected with HRP‐conjugated goat‐anti‐rabbit antibodies followed by ECL detection.

### 4.8 Pulse chase analysis

Pulse‐chase experiments were used to monitor intracellular transport of Gas1 and CPY and to monitor Sec23 and Sec24 degradation. These experiments were performed as previously described.^23^ Briefly, strains were grown to mid‐log phase at 30°C, starved for 15 minutes, and labeled for 5 minutes with 1 μL per OD of cells of EXPRESS ^35^S Protein Labeling Mix (*PerkinElmer*) for 5 minutes. The label was chased with excess rich media and 2 OD aliquots of cells harvested at different times. Cells were lysed in detergent and the protein of interest was immunoprecipitated from cell lysates, separated by SDS‐PAGE, and detected by phosphorimaging using a Typhoon scanner (*GE Healthcare*). The protein bands were quantified using Fiji and the percentage of the mature band in each sample was plotted with Prism 7.0 (*GraphPad Software*).

### 4.9 Fluorescence microscopy and FLIP assays

Sec13‐sfGFP imaging was performed in cells grown to mid‐log phase at 30°C in minimal media lacking tryptophan. Images were taken on a Nikon Ti2 with a 100×/1.49 NA Oil (TIRF) objective and a scientific complementary metal‐oxide‐semiconductor (sCMOS) camera.

FLIP was used to measure coat dynamics of Sec13‐sfGFP. Imaging was performed in cells grown to mid‐log phase at 30°C in minimal media lacking tryptophan. Images were taken on an Andor revolution spinning disk microscope with a 40×/1.3NA oil immersion objective and a EMCCD camera. Images were collected using the Andor iQ3 software. A small region of interest was repeatedly photobleached, and the fluorescence intensity of the whole cell was measured for a loss of signal, representing protein that had diffused into the bleaching area. Fluorescence intensity was measured using Fiji and statistical analysis was performed with Prism 7.0 (GraphPad Software).

## CONFLICT OF INTEREST

The authors declare no potential conflict of interest.

## AUTHOR CONTRIBUTIONS

Conceptualization: Elizabeth A. Miller; Investigation: Natalia Gomez‐Navarro; Software: Jérôme Boulanger; Writing ‐ original draft: Natalia Gomez‐Navarro, Elizabeth A. Miller; Writing ‐ review and editing: Jérôme Boulanger, Natalia Gomez‐Navarro, Elizabeth A. Miller; Funding acquisition: Elizabeth A. Miller.

5

## Supporting information


**Supplementary Figure 1** UPR activation in single mutant strains. Exponentially growing wild‐type and mutant cells carrying a *UPRE*‐*LacZ* reporter gene were lysed, and ß‐galactosidase activity measured using ONPG as a substrate. Error bars depict mean and SD of at least 3 independent experiments.

**Table S1**

**Table S2**

**Table S3**

## Data Availability

All data is available in the manuscript or the supplementary materials; plasmids and strains described can be obtained from EAM.
